# Recurrent Diarrhea Due to Cystoisospora belli in a Patient With HIV: A Case Report

**DOI:** 10.7759/cureus.103050

**Published:** 2026-02-05

**Authors:** Ana Victoria Gaxiola Ortiz, Jorge Arturo Valdivieso-Jimenez, Jairo R Villanueva-Toledo

**Affiliations:** 1 Infectious Diseases, Hospital Regional de Alta Especialidad de la Península de Yucatán IMSS Bienestar, Merida, MEX; 2 Internal Medicine, Hospital Regional de Alta Especialidad de la Península de Yucatán IMSS Bienestar, Merida, MEX; 3 Investigation, Hospital Regional de Alta Especialidad de la Península de Yucatán IMSS Bienestar, Mérida, MEX; 4 Investigation, Secretaría de Ciencia, Humanidades, Tecnología e Innovación Secihti (SECIHTI), México, MEX

**Keywords:** clotrimoxazole, cystoisospora belli, diarrhea, highly active antiretroviral therapy (haart), human immunodeficiency virus (hiv), immune recovery

## Abstract

*Cystoisospora belli* (*C. belli*) is an opportunistic protozoan that primarily affects immunocompromised individuals, including people living with human immunodeficiency virus (HIV). Although the widespread use of antiretroviral therapy (ART) and trimethoprim/sulfamethoxazole (TMP-SMX) prophylaxis has significantly reduced its global incidence, cases continue to be reported among individuals with inmune reconstitution. The pathogen remains a significant cause of chronic diarrhea, dehydration, and malabsorption syndrome in Latin America, contributing to increased morbidity and mortality. We present the case of a 27-year-old cisgender man living with HIV who experienced recurrent episodes of diarrhea due to *C. belli*, despite good adherence to ART and a CD4 count >200 cells/mm³. Initial parasitological stool examinations yielded negative results; however, a gastrointestinal multiplex polymerase chain reaction (PCR) panel subsequently identified *C. belli*. The patient received several courses of TMP-SMX with only partial improvement and requires ongoing secondary prophylaxis.

## Introduction

*Cystoisospora belli *(*C. belli*)-associated diarrhea occurs predominantly among immunocompromised individuals, including people living with human immunodeficiency virus (HIV), increasing the likelihood of recurrent disease and related complications [1]. Transmission occurs via the fecal-oral route, generally through the consumption of contaminated water or food. During the life cycle of *C. belli*, sporocysts excyst in the intestine and release sporozoites that remain confined to the epithelium of the intestine, bile ducts, and gallbladder [2]. The introduction of highly active antiretroviral therapy (HAART) in recent years has strengthened immune response capacity and, together with primary prophylaxis using trimethoprim and sulfamethoxazole (TMP-SMX), has contributed to reducing opportunistic infections such as *C. belli* [3]. In this report, we describe the case of a person living with HIV who experienced recurrent *C. belli* infection after initiating antiretroviral therapy (ART).

## Case presentation

We present the case of a 27-year-old cisgender male living with HIV, diagnosed in 2023 (stage C2, baseline CD4 count 167 cells/mm³). He was receiving antiretroviral treatment with bictegravir/emtricitabine/tenofovir alafenamide, maintaining adequate adherence. Despite achieving an undetectable viral load and immune reconstitution (current CD4 count 350 cells/mm³), the patient experienced chronic diarrhea persisting since his HIV diagnosis. These episodes occurred approximately three times per month, were managed symptomatically with pancreatin and simethicone with only temporary remission, and occasionally required hospitalization for dehydration.

Recently, the patient was prescribed cholestyramine for presumed bile acid malabsorption; however, this paradoxically exacerbated the diarrheal symptoms, leading to severe volume depletion and referral to the Infectious Diseases service. Upon admission to Internal Medicine, the patient presented with severe dehydration and acute kidney injury. Cholestyramine was immediately discontinued, and fluid resuscitation was initiated alongside empiric management with levofloxacin.

Initial diagnostic interrogation via serial stool ova and parasite (O&P) examinations and stool cultures yielded negative results. History revealed that during a prior hospital stay at a community facility, the patient had received multiple antibiotic courses including quinolones, cephalosporins, metronidazole, and TMP-SMX, which resulted in only partial clinical improvement. Given the high clinical suspicion and persistent symptoms in an immunocompromised host, a gastrointestinal PCR (polymerase chain reaction) panel was ordered. The panel identified *C. belli.*

Targeted treatment was initiated with oral TMP-SMX 160/800 mg every 12 hours for a planned duration of 21 days. However, the treatment course was complicated. During the initial phase (day 10 of therapy), the patient developed a severe recurrence of diarrhea resulting in significant dehydration, necessitating readmission. He was transitioned to intravenous TMP-SMX 160/800 mg every 12 hours for seven days, with clinical improvement noted by day three. Oral therapy was subsequently resumed.

During this hospitalization, *Clostridioides difficile *(*C. difficile*) toxin testing returned positive. Metronidazole 500 mg every eight hours was prescribed (as vancomycin was unavailable). However, the patient did not take the metronidazole yet continued TMP-SMX, and interestingly, the diarrhea did not recur at that time.

Five days following discharge, diarrheal episodes recurred despite adherence to the standard TMP-SMX regimen. Consequently, the TMP-SMX dosage was escalated to 160/800 mg every six hours for 14 days. Due to the previous equivocal *C. difficile* result, vancomycin 125 mg orally every six hours for 10 days was added empirically; repeat toxin testing was negative. Following the completion of the high-dose regimen, prophylaxis with TMP-SMX 160/800 mg every 24 hours was attempted.

Over the past six months, the clinical course has been unstable, with the patient experiencing approximately three episodes of diarrhea per month. These episodes required hospitalization for dehydration and intravenous treatment on three separate occasions, treated with four-week courses of TMP-SMX for each relapse. Consequently, the management strategy was adjusted to indefinite secondary prophylaxis. The patient is currently receiving TMP-SMX 160/800 mg every 24 hours indefinitely to suppress recurrence.

## Discussion

*C. belli* (formerly* Isospora belli)* is a parasite that primarily affects immunocompromised individuals, including people living with HIV, particularly in regions with tropical and subtropical climates. In Latin America, the organism has been documented in Mexico, several Central American countries (including El Salvador), and multiple South American countries (such as Peru, Bolivia, Venezuela, Brazil and Argentina), with higher prevalence in under-resourced areas with limited access to sanitation and drinking water [4]. Most people living with HIV who develop cystoisosporiasis have a CD4 count below 250 cells/mm³ and a history of current or past residence in a high-prevalence geographic region [5]. In contrast with typical presentations, the person described in this case initiated TMP-SMX prophylaxis soon after the HIV diagnosis; however, diarrhea recurred whenever the medication was transitioned from a therapeutic regimen to prophylactic dosing, despite an undetectable viral load and a CD4 count above 200 cells/mm³.

A study conducted in Los Angeles, USA, followed 16,351 people living with HIV for eight years and reported a *C. belli* prevalence of 1%. The highest prevalence occurred among individuals born in El Salvador (7.4%), Mexico (5.4%), or other Hispanic groups (2.9%) [1].

Transmission occurs through ingestion of water or food contaminated with fecal matter containing sporulated oocysts. After ingestion, the oocysts invade the mucosa of the small intestine-particularly the enterocytes-where they multiply and induce symptoms associated with chronic diarrhea. C. belli completes its life cycle within the intestine, producing immature, unsporulated oocysts approximately nine to 17 days after infection. These oocysts are excreted in the feces, and sporulation occurs externally in the environment over approximately 24 hours to 10 days. Once sporulated, the oocysts become infectious and can survive for extended periods [6].

Common symptoms of *C. belli* infection include watery, non-bloody diarrhea, vomiting, fever, abdominal pain, weight loss, electrolyte abnormalities, and, in some cases, eosinophilia74 [7]. In this case, the person experienced all of these symptoms except fever. Mild eosinophilia (610 cells/µL) was documented once; during subsequent hospitalizations, eosinophil counts remained below 500 cells/µL.

Beyond chronic diarrhea, extraintestinal manifestations have been described in people living with HIV, including severe cachexia, erythematous and hemorrhagic lesions in the small intestine, ulcerations up to 5 mm in diameter, brush-border atrophy and mesenteric lymphadenopathy. These findings have been linked to coinfections such as* Pneumocystis jirovecii*, cytomegalovirus, and candidiasis [4].

The diagnosis of *C. belli* infection is usually established in immunocompromised individuals with compatible gastrointestinal symptoms through the detection of oocysts in stool samples. Because oocyst excretion may be intermittent as seen in other parasitic diseases serial stool examinations may be required when clinical suspicion persists. When identified, *C. belli* oocysts typically have thin walls and an ellipsoidal shape, measuring 23-36 µm by 12-17 µm. Consequently, modified acid-fast techniques or ultraviolet fluorescence microscopy are needed for proper visualization [8].

Management of *C. belli* infection involves supportive care, including aggressive hydration, electrolyte replacement and nutritional support. In cases of persistent diarrhea or malabsorption, parenteral nutrition may be necessary until oral intake is tolerated. Chronic diarrhea can lead to fat and vitamin malabsorption, manifesting as prolonged prothrombin times and low albumin levels [9].

In immunocompetent individuals, the illness often resolves spontaneously within five to seven days. If symptoms persist, TMP-SMX for seven to 10 days is typically curative [10]. Immunocompromised individuals with symptoms should receive treatment. The preferred regimen is TMP-SMX 160/800 mg twice daily for seven to 10 days, although some guidelines recommend higher doses or treatment extension up to four weeks depending on clinical response. After completing therapy, secondary prophylaxis is recommended until the CD4 count remains above 200 cells/mm³ for at least six months [11].

Emerging literature from developing countries documents persistent *C. belli* infection despite effective treatment and immune reconstitution. Clinical outcomes range from recovery to chronic diarrhea or mortality. Several pathophysiological mechanisms have been proposed to explain this persistence, including endogenous reinfection (where oocysts sporulate within the host) and the reactivation of monozoic tissue cysts from extraintestinal sites (2). Furthermore, recurrence may be driven by impaired mucosal immunity (specifically CD4+ and Th17 T-cell defects) despite ART, antimicrobial resistance, re-exposure via contaminated sources, or drug malabsorption secondary to intestinal inflammation [12].

## Conclusions

Diagnosing *C. belli* infection remains challenging among people living with HIV, as diarrhea may have multiple etiologies and oocyst shedding is often intermittent, resulting in negative stool results even in active disease. Although many diagnostic methods exist, in our environment they are not always accessible and generally require trained personnel and special stains for identification. Therefore, the PCR test is a valuable tool for diagnostic confirmation, offering a high sensitivity (90-100%) and specificity (98-100%).

Some individuals may continue to experience chronic diarrhea despite ART and CD4 counts above 200 cells/mm³. In such cases, prolonged or even indefinite continuation of treatment and secondary prophylaxis may be necessary.
